# Monkeypox with genital ulcer as the first symptom

**DOI:** 10.11604/pamj.2024.47.108.42950

**Published:** 2024-03-05

**Authors:** Jianping Zhou, Linna Lv

**Affiliations:** 1 Linping Campus, The Second Affiliated Hospital of Zhejiang University School of Medicine, Hangzhou, China

**Keywords:** Monkeypox, monkeypox virus, genital ulcer

## Image in medicine

A 35-year-old male patient presented to our clinic in early December 2023 with superficial painless ulcers on the genital for one month. After questioning him, we learned he had high-risk homosexual behavior recently. The examination confirmed that his human immunodeficiency virus (HIV) status was positive, and the serological test for syphilis was negative. But 5 days later, his condition deteriorated rapidly, the genital ulcer turned so painful that he could not sleep. At the same time, he had a high fever, muscle soreness, and headache. His physical examination showed obvious swelling of the penis, and there were multiple regular circular ulcers with a white peripheral border and purplish-red central bottom on the glans penis. Ulcerative sores were present in his oral cavity. In addition, red papules appeared on his face, palms, soles, and back, and were accompanied by a bilateral enlarged inguinal lymph node. With these findings, we confirmed monkeypox virus infection through real-time PCR in skin lesions of the glans penis finally. The patient received supportive care and took cefuroxime orally by himself. Twenty-three (23) days later, the symptoms disappeared completely.

**Figure 1:**
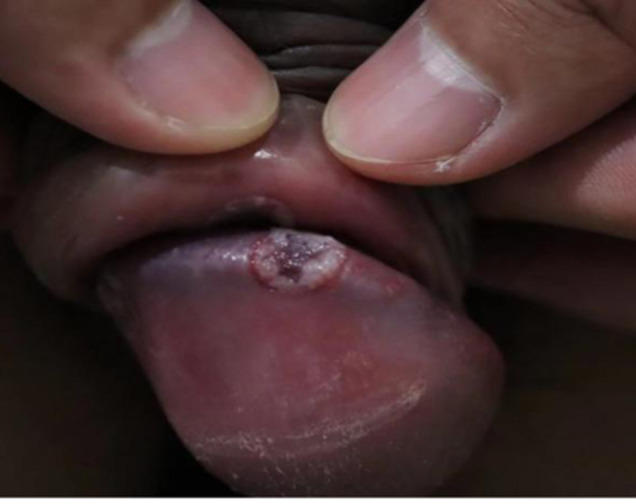
hydrophallus, multiple regular circular ulcers with a white peripheral border, and purplish red central bottom on the glans penis

